# Identifying Optimal Models to Represent Biochemical Systems

**DOI:** 10.1371/journal.pone.0083664

**Published:** 2014-01-08

**Authors:** Mochamad Apri, Maarten de Gee, Simon van Mourik, Jaap Molenaar

**Affiliations:** 1 Biometris, Wageningen University, Wageningen, The Netherlands; 2 Netherlands Consortium for Systems Biology, Amsterdam, The Netherlands; 3 Industrial and Financial Mathematics Group, Bandung Institute of Technology, Bandung, Indonesia; University Paris South, France

## Abstract

Biochemical systems involving a high number of components with intricate interactions often lead to complex models containing a large number of parameters. Although a large model could describe in detail the mechanisms that underlie the system, its very large size may hinder us in understanding the key elements of the system. Also in terms of parameter identification, large models are often problematic. Therefore, a reduced model may be preferred to represent the system. Yet, in order to efficaciously replace the large model, the reduced model should have the same ability as the large model to produce reliable predictions for a broad set of testable experimental conditions. We present a novel method to extract an “optimal” reduced model from a large model to represent biochemical systems by combining a reduction method and a model discrimination method. The former assures that the reduced model contains only those components that are important to produce the dynamics observed in given experiments, whereas the latter ensures that the reduced model gives a good prediction for any *feasible* experimental conditions that are relevant to answer questions at hand. These two techniques are applied iteratively. The method reveals the biological core of a model mathematically, indicating the processes that are likely to be responsible for certain behavior. We demonstrate the algorithm on two realistic model examples. We show that in both cases the core is substantially smaller than the full model.

## Introduction

Biochemical networks are often very complex. The complexity may arise from the large number of components involved in the network and/or from their intricate interactions. When such systems are modeled by differential equations, we obtain a large non-linear differential equation system with many parameters. There are some advantages for having a large model, e.g., it may capture in detail the mechanisms of the system and therefore might give accurate predictions. On the other hand, model complexity also gives rise to severe problems, e.g., hard understanding of system behavior under varying conditions; long computing times, especially in case of stiff models; and parameter identification problems, especially in the case of limited data availability. To overcome these issues, reduced models that still capture the essential features of the system are highly desirable.

Several methods for model reduction are already available, e.g., time-scale separation [1]–[4], sensitivity analysis [5]–[7], and lumping [8], [9]. These methods typically require prior knowledge of the parameter values of the model before they can be applied. Therefore, only the first two above-mentioned problems might be remedied in this way, whereas the problem of parameter identification, which is often the most problematic issue in systems biology, remains. In addition, some of these methods may lead to reduced models that are structurally different from the original one. This is because a component in the new reduced model may be a combination of several components in the original model, or a component in the original model could be contained in several components of the reduced model. This usually obstructs the biological interpretation of the reduced model.

In previous work we developed a reduction method to simplify biochemical models in systems biology [10]. This method is based on the so-called “admissible region” concept, i.e., the set of parameters for which the mathematical model yields some required output. This concept reflects the parameter uncertainty that commonly occurs in systems biology models. In contrast to the methods mentioned above, our method does not require prior knowledge of the parameter values. It also does not require a transformation, so that the reduction result is directly interpretable. However, our procedure to construct a reliable reduced model was not yet complete. The method only makes use of data which were obtained from experiments under specific conditions. The behavior of the system under conditions that are different from these experiments, might not be well predicted.

In this paper we repair this shortcoming by presenting a novel approach to extract a reliable reduced model from a full model under a large variety of experimental conditions. The proposed approach combines a reduction method and a model discrimination method. By combining these two methods, we arrive at a simpler model that still has powerful prediction capabilities. This in turn will help us in understanding the behavior of the complex system, since such a reduced model apparently contains the core of the mechanisms underlying the system dynamics.

## Materials and Methods

Consider a biochemical network for which the dynamics of its 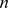 components is modeled by a system of ordinary differential equations (ODEs)
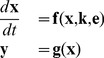
(1)with initial values
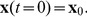
(2)Here, 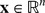 represents the concentration of the species in the network, 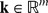 is the parameter set in the model, 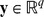 stands for the model output with 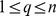, and 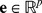 represents the experimental conditions under which the model output 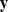 is measured. Throughout this paper, the components of 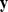 are referred to as “the target components” of the system. The measured data for 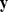 are denoted by 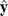.

**Figure 1:**
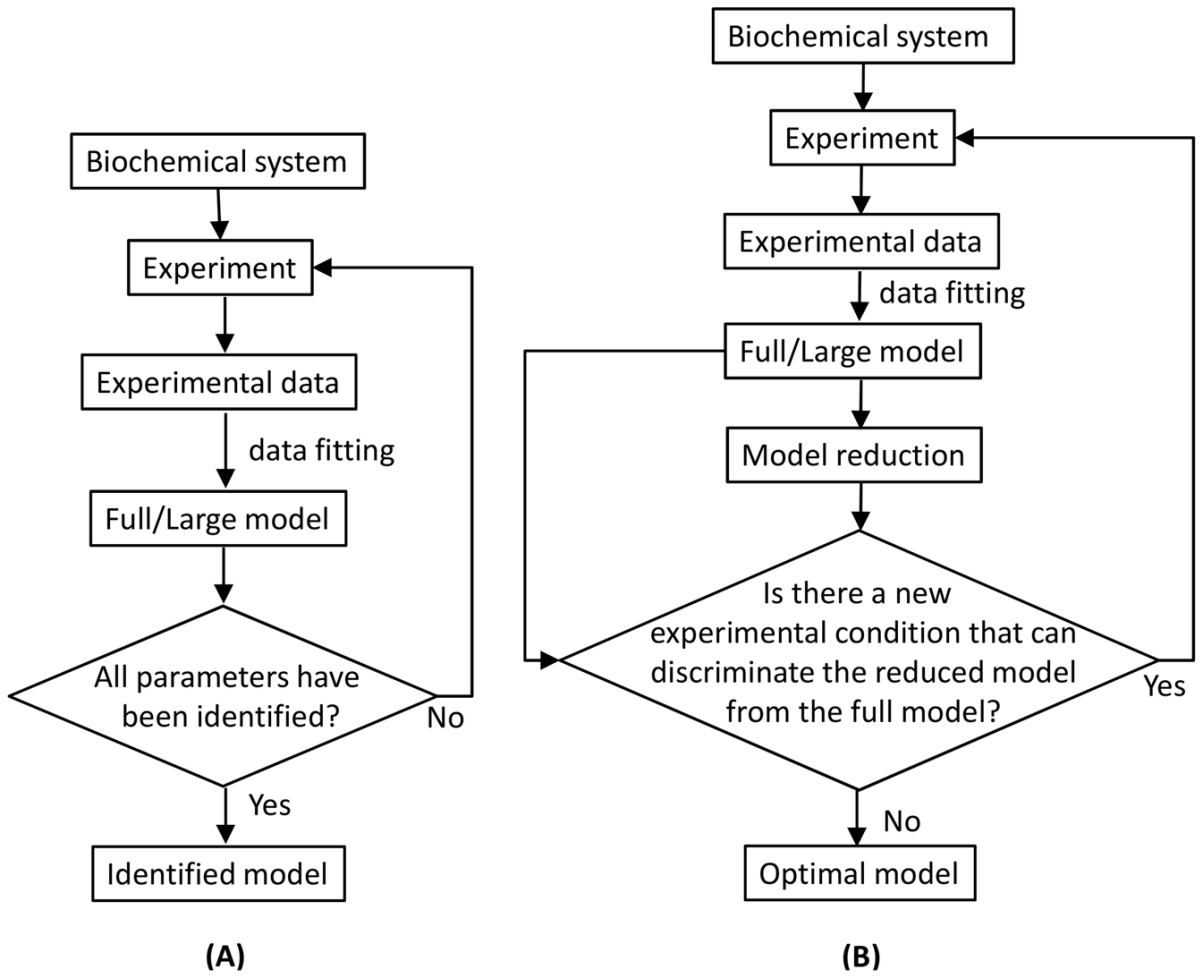
Approaches to estimate parameter in Systems Biology. (A) Common approach, (B) Proposed approach to yield optimal model with fewer parameters.

In practice, a common approach to estimate the parameter set 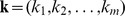 is by fitting model (1)–(2) to an initial dataset 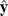. In this stage, the dataset that we have is usually very poor and thus the parameters that are found are not yet well identified, i.e., their values are yet rather sloppy. Therefore, a new experiment, based on optimal experimental design, is then carried out to obtain a new dataset and the parameter estimation is repeated. These steps are applied iteratively until all parameters can be hopefully identified, as depicted in Figure 1A. Unfortunately, in most cases, it is very difficult to identify all of them. This especially happens if the number of parameters is large. In those cases, it is convenient to work with a simpler model with less parameters so that parameter identification can be carried out efficaciously.

Although a reduced model contains less components and/or parameters than the original model, it is important that it should still be able to reliably predict the behavior of the system for *any feasible* experimental conditions that are considered relevant to answer questions at hand. Only in this case, the reduced model can replace the full model and fully represent the system. Thus, for example, if the initial condition of a particular biochemical species 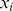 in the experiment can be in the range of 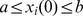, then the behavior of the system should be well predicted by the reduced model for any initial condition 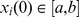. Also, if a particular perturbation can be applied in an experiment, e.g., deletion and/or knock-out of some genes, the behavior of the perturbed system should still be well predicted. In this paper, the set of all feasible experimental conditions is denoted by 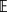. A reduced model that does not contain redundant component and/or parameter and can reliably predict the dynamics of the target components for any 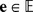 is referred to as “an optimal model”.

To extract an optimal model, we combine our reduction method [10] with a model discrimination method. The procedure is sketched in Figure 1B. The essence of this scheme is that for the obtained reduced model, it is investigated whether an experimental condition can be found for which the reduced model yields an outcome that is significantly different from what the full model would predict. If this is the case, the reduced model is not accepted as being “optimal”.

Notice that parameter estimation is frequently used in our procedure. For this aspect a vast body of separate literature exists, e.g., [11]–[16]. In our calculation, we made use of the nonlinear least square solver from MATLAB which is a local optimization algorithm.

### Model reduction

After having obtained a parameter estimate 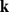 for the full model, the first step to obtain an optimal model is to reduce the model complexity by removing redundant components and/or parameters that do not contribute to the dynamics of the target components. For this purpose, we have developed a reduction method in [10] that utilizes the concept of admissible region. In this method, the parameters that are removed are those that are badly identified. The method does not necessarily require prior biological knowledge. However, the method can easily be tuned to incorporate prior knowledge, if this is available. The main features of our reduction method are summarized below.

#### Admissible region

Suppose that 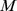 time-series data of the target components 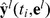 are obtained from experiments, which were conducted under 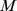 different experimental conditions 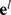, with 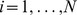, and 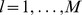. We measure the distance of the model output (target components) to the time-series data using the following function
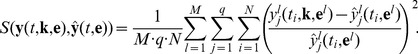
(3)Notice that 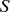 is the least squares measure that can be interpreted as the average squared deviation between the model prediction and the data.

Let us introduce a tolerance 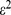 which indicates how much difference we accept as discrepancy between the data and the model prediction. In many cases, the variance of the noise from the experimental data can be used as a guidance to choose a suitable value for 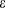. Then, any parameter vector 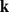 such that
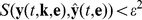
(4)is acceptable to represent the parameters of the system, since it is capable of producing the dynamics within the required accuracy. We say that all parameter vectors 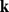 that satisfy (4) constitute the so-called “admissible region” (AR). Thus,
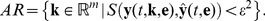
(5)Notice that the region 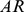 reflects the parameter sloppiness in the model, i.e., different parameter sets may yields the same model dynamics [17]. Additionally, the broad admissible region implies that the model encounters a practical identifiability problem [18].

#### Reduction method

Since all parameter vectors in the admissible region yield an acceptable dynamical behavior of the system, the shape of the 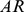 may suggest whether reduction is possible. For example, if the admissible region includes a part of a parameter axis, then this parameter can apparently be set to zero and could thus be excluded from the model. If the region extends to infinity in a certain parameter direction, then some terms or state-variables in the ODEs might be lumped. This analysis may thus lead to a simpler representation of the dynamics of the biochemical system.

Describing the admissible region and deducing the possible reductions is relatively easy for a small system, as shown in [10]. However, applying such analysis to a model with many parameters, which is typically the case in systems biology, can be very complicated. Fortunately, we notice that in practice it is not necessary to construct the admissible region completely. If one (or several) parameter(s) can be set to zero (or infinity) and the others can be re-optimized such that the resulting parameters 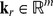 are still in the admissible region, then the model can be simplified. This reduction procedure can be carried out in a systematic way by applying first node reduction, then parameter reduction, and finally node lumping, as we will shortly discuss below.

#### Node reduction

First, we try to remove redundant nodes, one at a time. Here, e.g., node 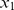 can be removed from the system if it can be eliminated in all equations and the parameters can be re-optimized such that (4) is satisfied. This procedure is repeated for 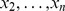. If one or more nodes have been removed, we cycle again through the remaining nodes and repeat the procedure until no further nodes can be removed.

#### Parameter reduction

To see whether a parameter, 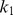 say, can be removed, we simply set 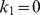 and re-estimate the other parameters. If (4) is satisfied, then indeed 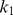 can be removed from the model. Next, this procedure is repeated for 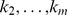. If one or more parameters have been removed, we cycle again through the remaining parameters and repeat the procedure until no further parameters can be removed.

Since the approach is heuristic, the result of the reduction might depend on the parameter ordering and might be not unique. In principle, all reduced models obtained this way are acceptable. However, for reasons of parsimony, the strongest reduction is preferable. For this purpose, we order the parameters based on the sensitivities
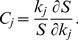
(6)Although these parameters sensitivities are local quantities, we found that in general they give a very good reduction rate, see [10].

#### Lumping

If a parameter that represents the strength of a reaction can be set at a very large value and the others can be adjusted to satisfy (4), it may indicate that the corresponding reaction can be considered as instantaneous. This implies that the two corresponding nodes that are connected by the reaction can be lumped, and hence may be replaced by one node. The procedure for lumping essentially follows the same steps as mentioned under parameter reduction.

### Model discrimination

Suppose that from the model reduction procedure above, we obtain a reduced model
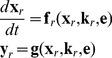
(7)where 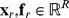 with 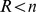, and 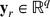 denotes the dynamics of the target components of the reduced model. Thus, we now have two different models to describe the measured target components, i.e., the full model and the reduced model. The next step is to investigate whether the reduced model will generate the same prediction as that of the full model for any feasible experimental conditions that are considered relevant to answer questions at hand. Only if all these predictions agree sufficiently, then we conclude that the full model can be replaced by the reduced model.

To check the predictive power of both models, an optimal experimental design approach to discrimate the models is utilized [19]–[22]. Here, we look for an experimental condition 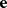 on 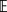 and time sampling 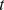 that maximize the distance between the full and reduced models in terms of the distance function 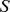 in (3). Mathematically, this can be written as
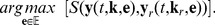
(8)


We say that a reduced model cannot be distinguished from the full model if their distance satisfies
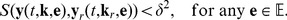
(9)with 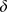 a value that denotes the tolerance criterion. The value for 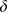 must be chosen by the modeller. Since 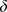 represents the worst deviation between the predictions of the reduced and the full models, the smaller the value of 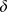 is, the more powerful the reduced model will be. However, 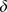 should be chosen larger than 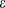, because otherwise we might end up with modeling noise.

### Model reduction and model discrimination applied iteratively

**Figure 2:**
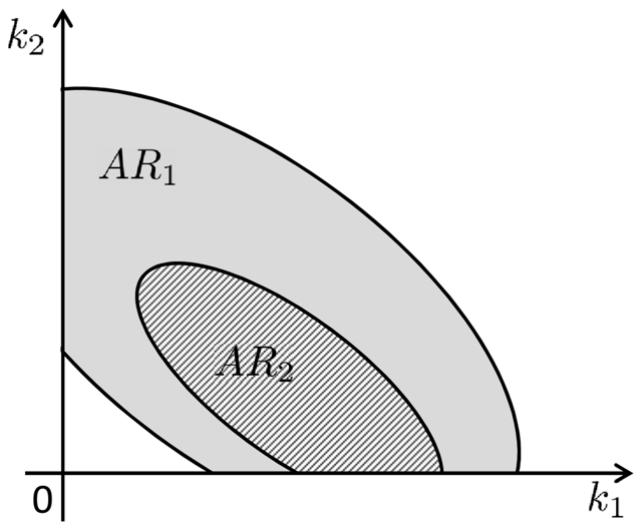
Illustration of an admissible region for a system with two parameters. Initially, the admissible region of the system is 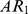. In this situation, a reduced model can be obtained either by setting 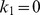 or 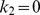. When a new dataset from a new experiment is incorporated, the admissible region shrinks to 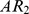. Thus, 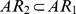. Now, a reduced model can only be obtained when 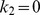.

Here, we discuss the essential features of our algorithm to obtain an optimal model. For illustrational purposes we sketch in Figure 2 the parameter space of a system with only two parameters. The admissible region shown in Figure 2 contains the parameter vectors for which the full model produces the required result, within the specified tolerance. Note that the candidates for the parameter set of the reduced model are those that lie on the parameter axes within the admissible region.

To find the optimal model, we could in principle compare each parameter set candidate of the reduced model in 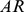 to the full model under all feasible experimental conditions. In practice, this is impossible. Therefore we apply an iterative algorithm. We first apply the reduction method in [10] and obtain a reduced model that at least for the measured target components shows the same behavior as the full model. Next, we compare this particular reduced model with the full model under all feasible conditions and select the condition for which the difference is biggest by applying hybrid optimization (a combination of global and local optimization). This optimization is carried out many times to make sure that the condition that we find is close to the global maximum. This is called “discrimination”.

Normally, this difference is still huge in this first step. Then, we add the data according to this new experimental condition to our dataset. In the second step this extended dataset is used as starting point. This second step starts with calculation of updated parameters 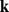 for the full model. Then, the reduction method from [10] is applied leading to a new reduced model. If this second reduced model is compared to the full model under all feasible conditions, one usually finds that the difference in model predictions becomes smaller than in the first step of the algorithm. The procedure is repeated until this difference between reduced model and full model is smaller than the threshold for all feasible experimental conditions. When the optimization procedure only yields conditions that make the deviation always less than our tolerance in (9), we accept that the reduced model approximates the full model everywhere on 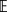. This resulting model for which this holds is called “optimal”.

### Algorithm

In summary, the method that we propose consists of the following steps:

1. Obtain data from experiment.
2. Estimate the parameters of the full model.
3. Apply reduction to the full model.
4. Try to discriminate the resulting reduced model obtained in step 3) from the full model obtained in step 2).
5. If there indeed exists an experimental condition that can discriminate them, add the data according to this condition to the dataset and repeat step 2)–4). Otherwise, an optimal model has been obtained.

## Results

In our view, model reduction and model discrimination should be an integral part of the modelling-experimental cycle. When model discrimination identifies an experimental condition to separate the reduced model from the full model, then the corresponding experiment should be carried out in the lab. However, in order to show how the proposed approach may work out in practice, here we use a different approach. The method is applied to two established models from literature: a flowering model that describes the genetic interactions underlying flower development, and an EGFR network model of a signaling transduction network. Both models have been published with a full parameter set, and in this sections we adopt the outcome of these models (with some additional noise) as experimental result. Note that we do not use these published parameters in our own fitting/reduction/discrimination algorithm. For simplicity, here we used the same sampling times 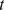 for all experiments. In practice, one might have different sampling times for each experiment. In this way, the choice of sampling times could be part of the experimental setup.

### Flowering network model

**Figure 3:**
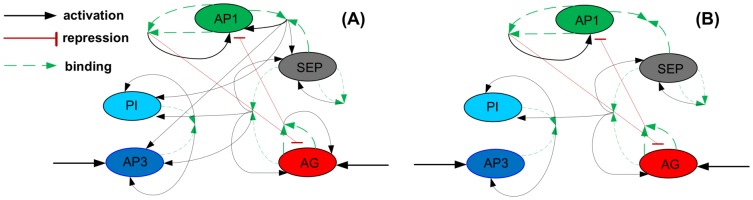
Graphical representation of the genes interactions of flowering in Arabidopsis. (A) The full model, (B) The reduced model.

The dynamic model from [23] describes the genetic interactions of five types of MADS genes underlying flower development. The expression patterns of these genes are associated with floral organ identity via the so-called ABCDE model [24]. Of the four floral organ types in Arabidopsis, sepals are linked to high expression of the A gene, petals with A and B, stamens with B and C, and carpels (including ovules) with C and D. All organs require high expression of the E gene. The genes that represent the five ABCDE functions in this model, are *AP1* (A), *AP3* (B), *PI* (B), *AG* (C), *SHP1* (D) and *SEP* (E). Gene expression is modeled as protein concentration, and the genes interact in this model via pairs of two proteins (dimers) that regulate each other's genetic transcription rate, see Figure 3. The network dynamics differentiates between the floral whorls via location-specific trigger mechanisms.

The model consists of 6 state variables (representing proteins and dimers), and 37 parameters representing the rates of the biochemical interactions (see supplements for details). The model is able to generate realistic predictions for the following experimental conditions
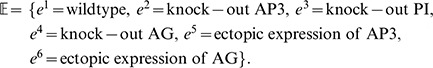
(10)For knock-out experiments, the initial and production terms of the corresponding genes are set to zero. For ectopic expression, we assume that the initial concentrations of the corresponding genes are available abundantly; therefore they are set to a high level. In this case, 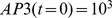 whereas 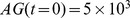. Thus, there are only six feasible experiments: wild-type experiments; setting initial and production term of AP, PI, and AG to zero; and setting initial concentration of AP3 and AG to a specific high value. These feasible experiments are taken from [23].

To show how our proposed method can be applied to a real biological system, we assume that the parameters in the model are unknown and have to be estimated from the experimental data. The six measured gene expressions are regarded as our target components in this example. We further assume that the measurements can be conducted at the conditions in (10). Then we investigate whether the structure of the proposed full model contains redundancy and hence, whether the dynamics can be described by a simpler model.

As experimental outcome, we generate the data of the target components using the full model and the parameters in [23], adding relative normal random noise of 20%. The measurements are assumed to be performed at 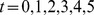 days.

**Figure 4:**
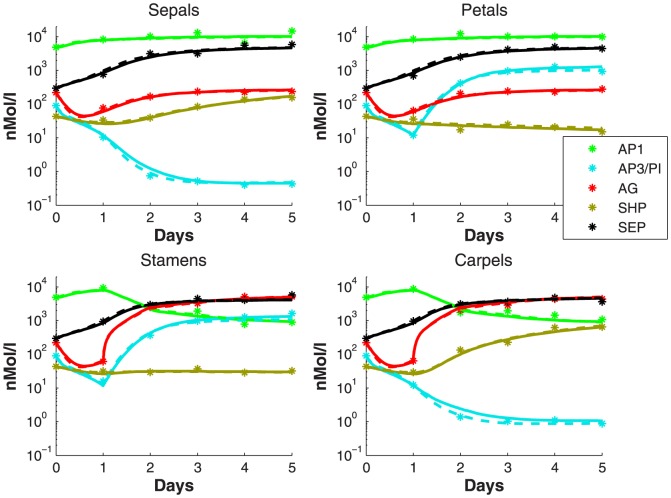
The concentration dynamics of the proteins. These proteins are part of dimer complexes in each of the four organ initiation sites for the wildtype dataset. The solid and dashed lines show that the dataset can be fitted by the full model with 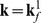 as well as by the reduced model with 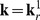.

For the initial dataset, the wild-type measurement is carried out. The results are shown in Figure 4, denoted by ‘*’. If we set 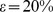 we found that the dataset can be well represented by the model with many possible parameter sets, one of them is 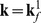. When reduction is applied, it turns out that 7 out of 37 parameters can be removed from the model and yet the reduced model can still fit the dataset as shown by the dashed lines in Figure 4. This parameter set is denoted by 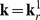.

**Figure 5:**
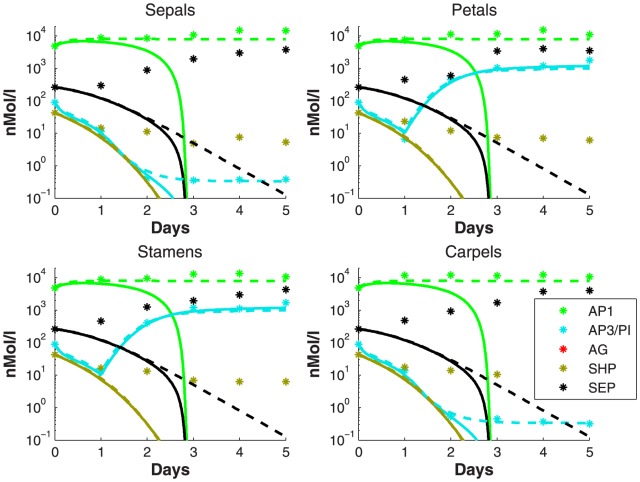
Model discrimination applied to the first full model and reduced model. Model discrimination finds that knocking out AG will distinguish the reduced model with 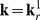 from the full model with 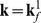. When an experiment is conducted for this mutant, we obtain the dataset that is denoted by ‘*’.

Thus, we have two possible models to represent the behavior of the wildtype, i.e., the full model and the reduced model. Following the algorithm described in the Methods section, model discrimination is applied to find which experiments from (10) can discriminate the two models. Setting the tolerance criterion in the model discrimination equal to 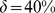, we find that experimental condition
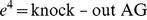
(11)distinguishes the reduced model from the full model, as shown in Figure 5. In this case, the distance between the reduced and the full models is 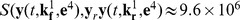. Therefore, this experiment is then carried out to confirm this difference, and we obtain the dataset denoted by ‘*’ in Figure 5.

From the new experiment, we found that the predictions from both the reduced and the full model cannot represent the behavior of the mutant. Therefore, the parameters in the full model should be re-estimated to fit both the wild-type and the mutant AG dataset and the reduction should also be repeated. Then, we obtain a new parameter set 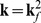 for the full model and a reduced parameter set 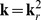. In this new reduced model, 6 parameters can be reduced.

Given the list of possible experiments in (10) and the threshold value 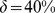 for model discrimination, we found that the new reduced model now cannot be discriminated anymore from the full model. Thus, we may claim that the reduced model can replace the full model to represent all system behavior that we are interested in. This is underpinned by the fact that all datasets that are obtained for all conditions in (10) can be predicted very well by the reduced model, as shown in Figure S1. The parameter values for the full and optimal models are shown in Table 1.

**Table 1:** Parameter values of the full and optimal models in the flowering model.

Parameter	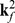	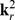	Parameter	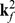	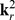
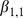	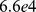	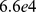	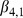	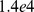	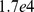
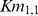	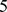	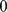	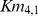	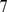	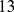
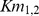	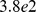	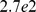	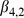	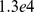	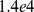
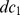	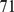	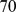	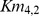	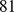	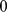
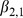	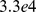	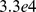	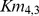	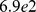	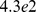
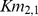	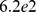	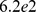	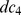	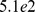	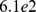
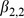	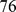	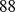	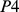	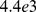	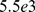
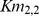	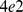	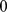	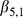	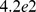	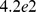
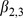	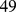	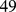	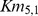	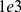	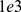
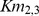	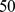	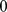	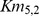	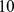	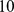
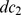	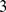	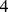	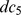	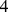	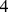
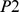	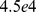	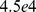	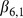	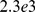	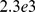
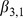	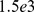	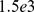	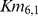	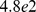	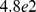
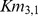	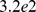	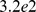	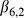	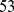	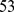
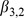	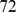	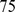	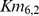	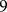	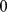
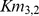	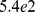	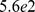	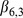	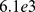	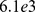
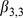	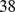	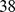	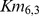	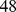	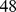
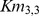	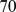	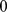	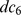	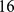	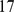
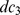	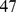	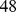			

The reduction allows for an interesting conclusion: all interactions that originate from dimer [AP1,SEP] are not needed to explain the behavior of the system under the conditions in (10). Thanks to reduction, we found that these interactions can be replaced by a constant production term. We conclude that the reduced model in Figure 3B is the core network that is responsible for the dynamics under conditions (10).

### EGFR model

**Figure 6:**
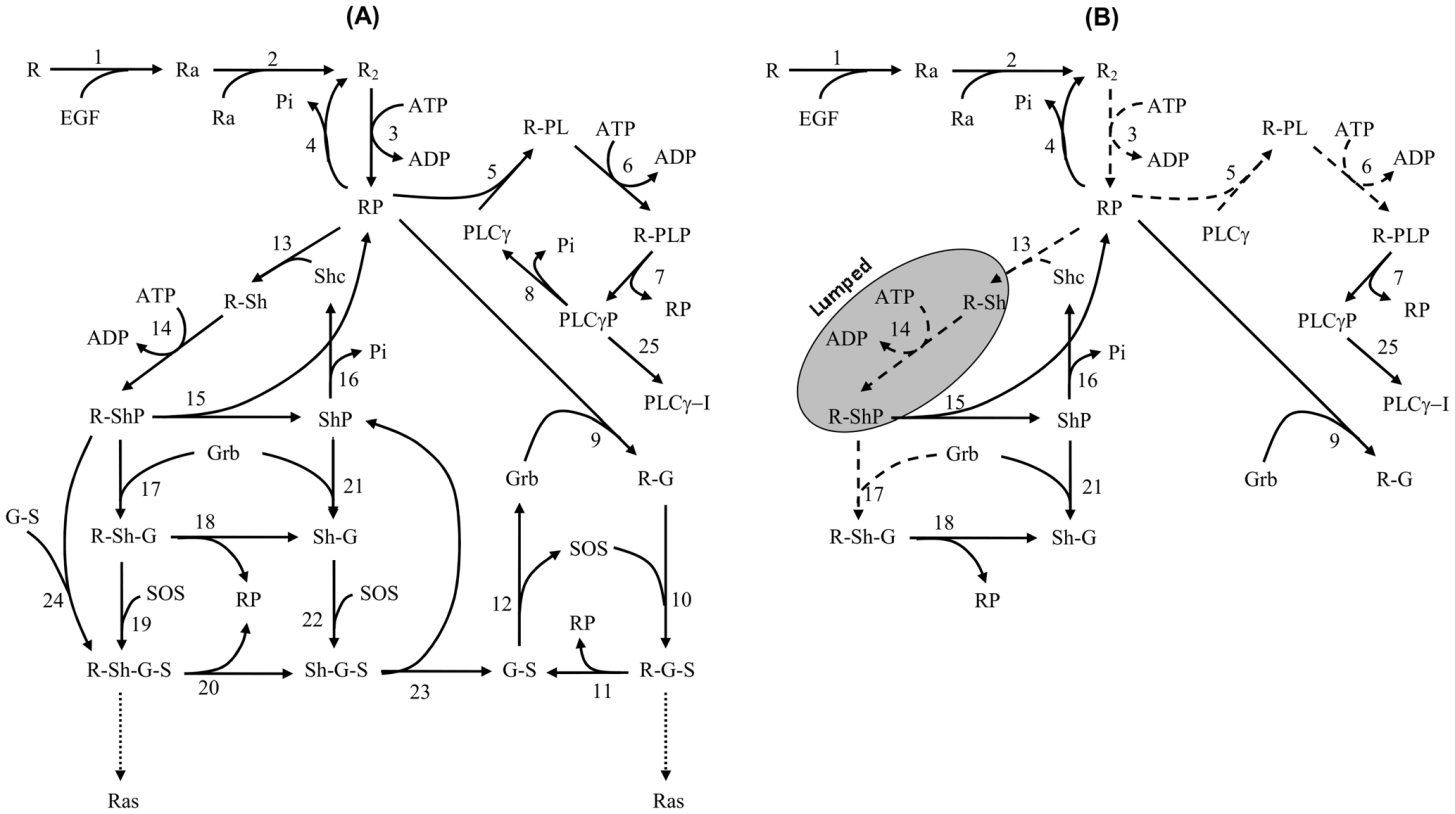
The EGFR biochemical network. A solid arrow represents a reaction with two kinetic parameters and a dashed arrow represents a reaction with one kinetic parameter. (A) The full network from [25], (B) The optimal network to produce the dynamics of the five target components for any experimental condition 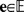 in (22).

Next, we apply our method to the epidermal growth factor receptor (EGFR) model from [25], of which the network is shown in Figure 6A. This model describes the cellular response to an epidermal growth factor (EGF) stimulation. The model consists of 23 biochemical components with 25 chemical reactions, described by ordinary differential equations (ODEs). This results in an ODE system with 23 state variables and 50 parameters. Since the kinetic scheme contains several cycles, the kinetic parameters involved in the cycles satisfy the so-called “detailed balance” relationships given by
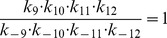
(12)

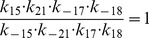
(13)

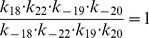
(14)

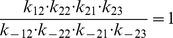
(15)

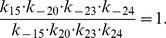
(16)The parameter values of the full model are given in Table 2 in [25].

To validate their model, the system was stimulated with different EGF stimulations (20 nM, 2 nM, and 0.2 nM) and the resulted transient response of several proteins were measured. These are the concentrations of phosphorylated EGFR, phosphorylated Shc, phosphorylated PLC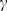, Grb2 bound in Shc, and Grb2 bound in EGFR, which are composed of several species in the model:
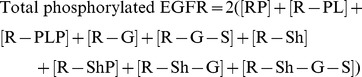
(17)

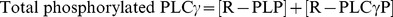
(18)

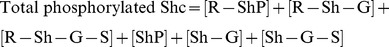
(19)

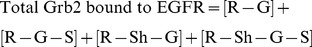
(20)

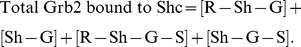
(21)


The model was then used to predict the dependency of the transient responses on the relative abundance of some signaling proteins, that is when the initial concentration of Shc was decreased by a factor of 4, the initial concentration of Grb2 was increased by a factor of 4, and when the initial concentration of EGFR was increased by a factor of 4.

To show how our method can be applied to this biological system, we assume that the parameters in the model are unknown and should be estimated from experimental data. The measured responses are regarded as our target components in this example. Since the target components were measured and predicted for different EGF stimulations and different initial conditions of EGFR, Shc, and Grb2, we assume that the relevant feasible experiments are to vary the stimulations and initial conditions of these species. Thus,
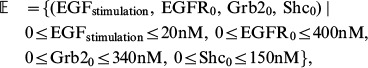
(22)where 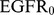, 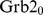, and 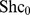 are the initial concentrations (concentrations at 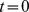) of 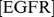, 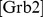, and 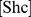 respectively. Notice that the space of feasible experimental conditions is very large. In model discrimination procedure, for this example, we utilize a hybrid optimization tool (combination of genetic algorithm and local optimization) in Matlab to obtain experimental conditions that maximizes the difference between the reduced and the full models.

As experimental outcome, we generate the data of the target components using the full model and the parameters in ([25]), adding a relative normal random noise of 5%. The measurements are assumed to be performed at 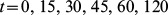 seconds.

For the initial dataset, we assume that it is obtained from experiments which are carried out at two different EGF stimulations, [EGF] = 20 nM and [EGF] = 2 nM. The other three initial conditions are set to [EGFR]_0_ = 100 nM, [Grb2]_0_ = 85 nM, and [Shc]_0_ = 150 nM. Thus, the initial dataset is obtained from the experiments with conditions
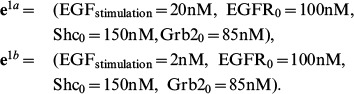
(23)


**Figure 7:**
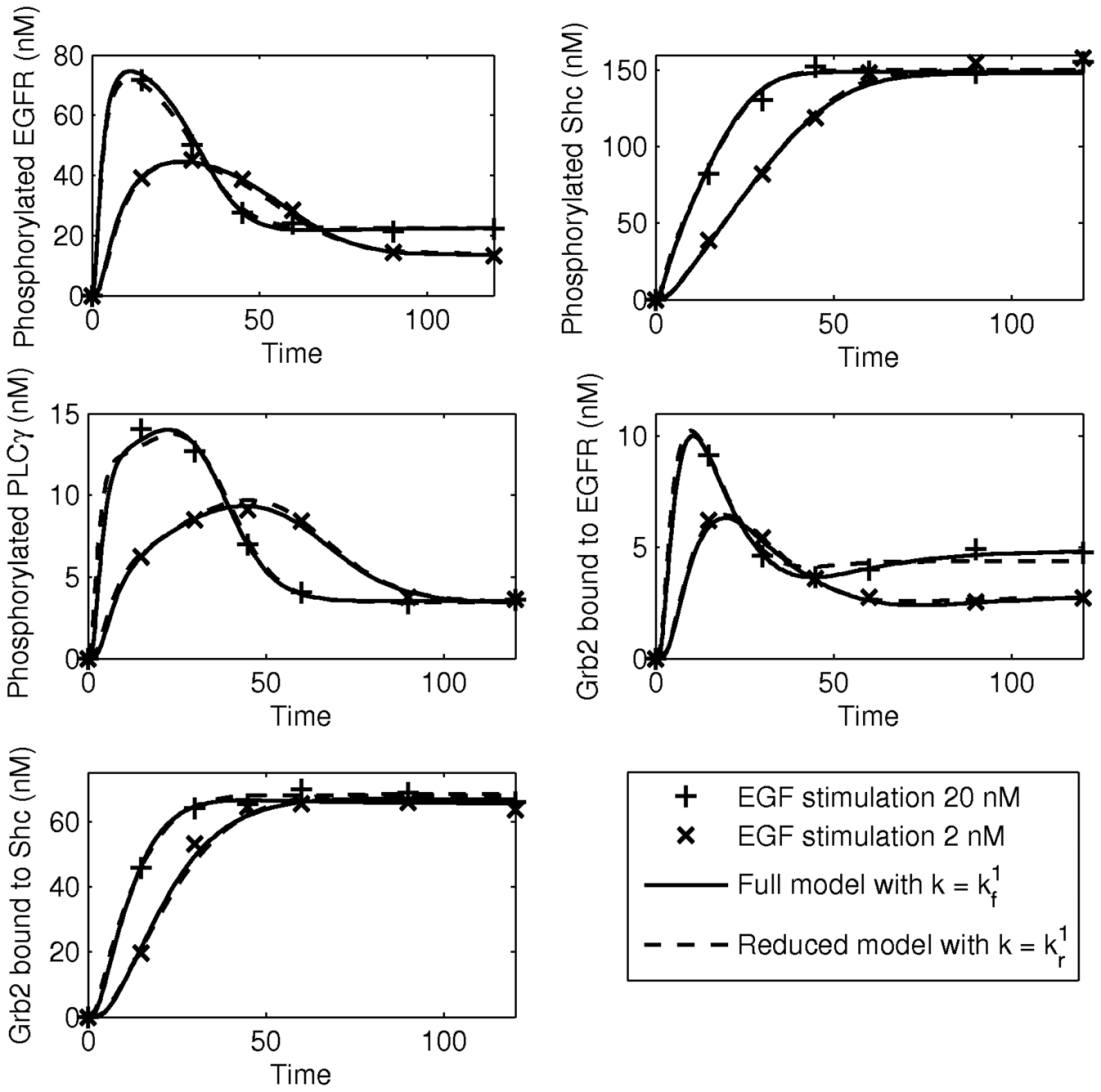
Dynamics of the target components for the start up dataset. The solid and dashed lines show that the dataset can be fitted by the full model with 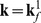 as well as by the reduced model with 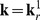.

The dynamics of the target components are shown in Figure 7, denoted by ‘+’ and ‘x’. If 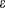 in (4) is set to 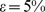 and parameter estimation is applied, we find that the dataset can be well represented by the model with many different parameter sets; one of them is 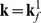. When reduction is applied, it turns out that 33 out of 50 parameters can be removed from the model. This parameter set is denoted by 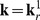. The reduced model can fit the dataset quite well, as shown in Figure 7.

Applying model discrimination, we find that with the experimental conditions
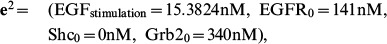
(24)the reduced model can be clearly distinguished from the full model, as can be seen in Figure S3. Their distance in this case is 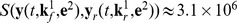.

To obtain an optimal model, we follow the procedure outlined in Section of Algorithm. First, experiment 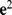 is performed to obtain a new dataset. So, the new dataset now consists of the combined dataset obtained with the experimental conditions 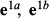 and 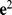. Parameter estimation and model reduction are now carried out to the combined dataset. This procedure yields 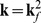 for the parameter set of the full model and 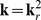 for the parameter set of the reduced model. The number of parameters that can be reduced turns out to be 31. Re-performing the model discrimination, we found that the experimental condition that maximizes the distance between the full model and the reduced model is now 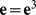 with distance 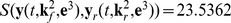. Setting the threshold value for model discrimination at 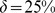, we find that after including four additional experimental conditions the reduced model with 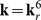 cannot be distinguished anymore from the full model with 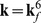. The network of the optimal model is shown in Figure 6B and the iterative process to obtain the optimal model is shown in Figure S4.

In the optimal model in Figure 6B, 24 parameters can be set to zero while one parameter, namely 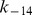, can be set to a very large value. The latter implies that the phosphorylation of [R-Sh] occurs very fast, and therefore, the components R-Sh and R-ShP can be lumped into one biochemical component in the optimal model. Thus, we end up with a model that consists of 17 biochemical components with 25 kinetic parameters. This result shows that we may remove six redundant components and 25 redundant parameters from the original model. The prediction for the five target components from the reduced model would then deviate at most 25% from that of the full model for *any* experimental condition in (22).

**Figure 8:**
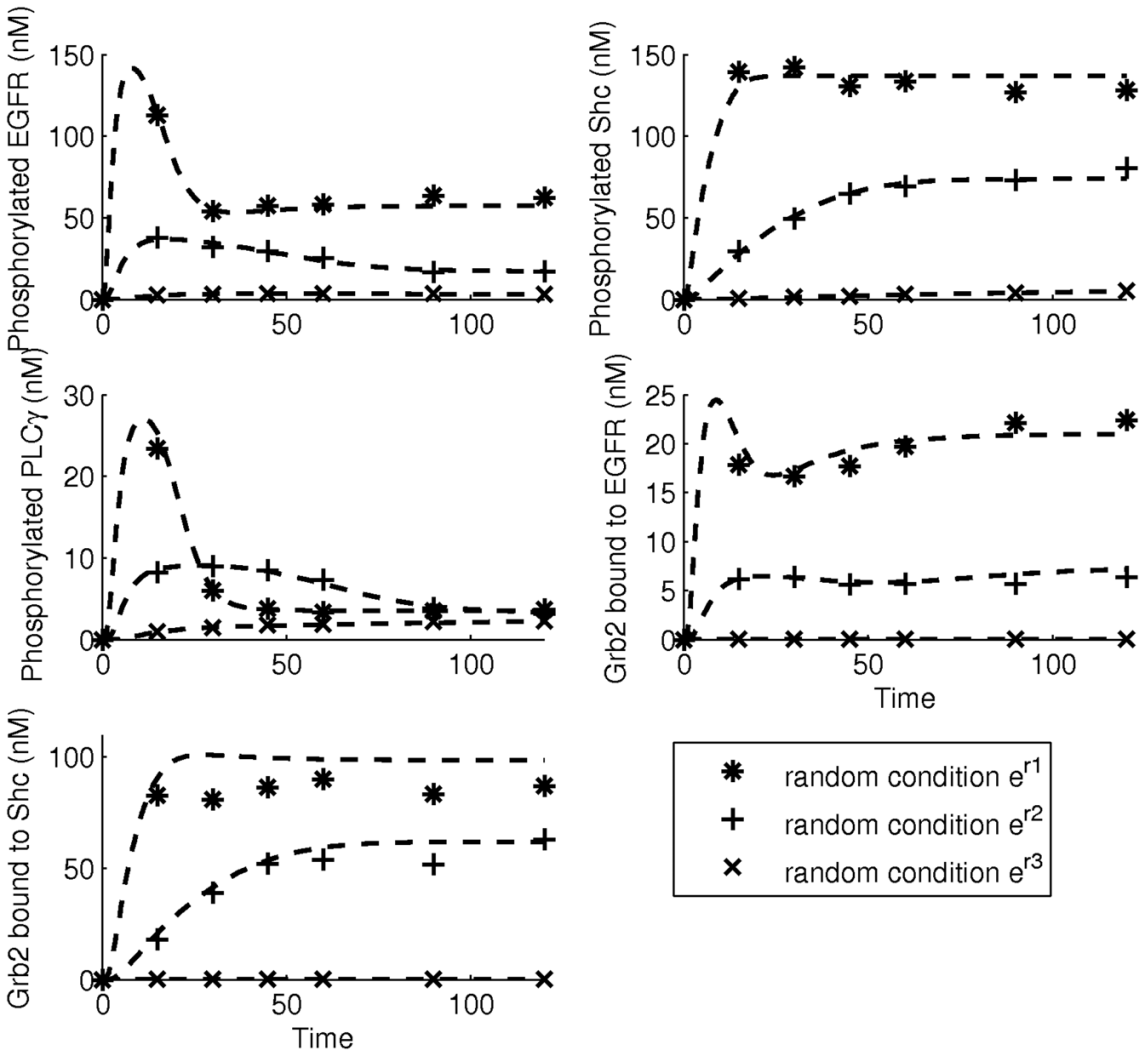
Validation of the optimal model with 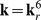. The data, marked with “*”, “+”, and “x”, are obtained from random experimental conditions 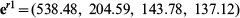, 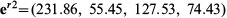, and 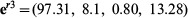 respectively. Predictions from the optimal model are indicated by the dashed lines.

As a validation, a number of experiments are performed with different random experimental conditions and the dynamics of the target components are predicted by the reduced model, as shown in Figure 8. The results show that the predictions of the reduced model are in very good agreement with the dynamics obtained from the experiments. Only in the first experiment, the prediction for Grb2 bound to Shc slightly deviates from the measurement. However, the deviation is still acceptable. We, therefore, conclude that the reduced model in Figure 6 B with parameter set 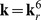 is an optimal model to produce the dynamics of the five target components, given the threshold value of 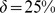. The parameters of the full and optimal models and the list of experimental conditions used to obtain the optimal model are shown in Table S1 and Table S2.

#### Ras pathway

The reduction results for the EGFR network allow for some nice conclusion. For example, a number of chemical reactions that lead to the activation of Ras protein via SOS are not longer preserved in the optimal model. Mathematically speaking, this implies that the parameters that are related to these reactions cannot be identified by only measuring the five target components above. From a biological point of view, deleting this branch is rather pointless since the function of EGFR is to activate the Ras→Raf→Mek→ERK cascade [26]. To preserve this chemical pathway in the optimal model, one should think carefully which complex protein(s) should be treated as additional target component(s), or alternatively, which constraint in the reduction should be taken into account. In other words, prior knowledge should help us to prevent undesired results. Fortunately, our method can easily be tuned to incorporate prior knowledge. In this specific case this could be done as follows.

We observe from the network in Figure 6A that one possibility to maintain the pathway to the Ras protein is by preventing one of the incoming reactions to R-Sh-G-S or R-G-S from elimination. In practice, this can be established by preserving one of the following parameters from being zero, namely 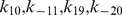 or 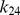. If we use this condition as our constraint in the reduction, we obtain the optimal model that is depicted in Figure S2C. Here, the activation of Ras protein can be achieved via R-Sh-G-S.

Also in this optimal model, parameter 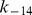 can be set to a very large value and thus R-ShP and R-Sh can be lumped into one biochemical component. The model contains 28 kinetic parameters, so about 44% of the kinetic parameters in the original model are redundant to represent the dynamics of the five target components. The iterative process to obtain this model is shown in Figure S5. As can be seen, the optimal model is now obtained after six new experiments have been performed. The parameters of the full and optimal models and the list of experiments to obtain the optimal model are shown in Table S1 and Table S3. Notice that in this reduced model, the parameters in the branch that was preserved by using prior knowledge are not identified very well from these experimental data.

## Discussion

In Systems Biology, we often face the problem of non-uniqueness: several models can describe measured data equally well. In such a situation one may sometimes choose between a model that includes a lot of details of the underlying mechanisms but is complex, very time consuming, and might be over-parameterized, or a reduced version that is much more convenient to handle, but might have less predictive power. When modelling a system that is a part of a large intricate environment, one should be careful in identifying components/interactions can give meaningful contributions to answer ones questions. It is tempting to add components, and thus parameters, to the model that are actually not required in governing the dynamics of the observed components. This may result in difficulty in identifying the parameter values, while at the same time the understanding of the key functionality may be obscured by superfluous details. Therefore, for the sake of understanding the system, speeding up the computation, and parameter identification, a simpler model is usually more favorable. However, since a simpler model contain less detailed mechanisms, its predictive power might be less reliable. Therefore, model reduction requires a careful approach.

At least two conditions must be satisfied by a reduced model to replace the full model. First, it should be able to fit the observed data; and second, it should have the same power as the full model to reliably predict the behavior of the system under different feasible experimental conditions that are considered important to answer questions at hand. The reduction technique in our approach assures the first requirement and the discrimination method ensures the last requirement. The model discrimination in our method can also be viewed as a way to verify whether the omitted components, reactions, and/or parameters in the reduced model give a meaningful contribution to the model prediction. If they do, the dataset from a new experimental condition will confirm this so that in the next reduction, the method cannot remove the corresponding components and/or parameters. The resulting model is a trade-off between reliability and simplicity: it does not contain redundant components, but has enough predictive power to reliably predict the behavior of the system. Thus, with the proposed method, the redundant components can be easily detected and removed so that at the end, our model only contains components and parameters that are essential in generating the required predictions of the system. Obviously, the optimal model contains the core mechanisms that underlie the behavior of the biological system.

Note that when we apply parameter estimation of the full model and model reduction for the first time, the initial data set may come from several experiments. Our example on EGFR model shows this (equation (23)) where the initial data were obtained from two experiments with different EGF stimulations. Obviously, one should make sure that the initial data set contains sufficiently rich information for parameter inference, but this is beyond the scope of our paper. After discrimination procedure, however, we do recommend to carry out only one single new experiment in every iteration, as it will be enough to falsify the result of the previous full model or reduced model. Furthermore, doing one experiment at a time will avoid doing experiments that are likely to be superfluous.

Identifying parameters with high accuracy is difficult. Therefore, in [17] it is suggested to focus on model prediction rather than on parameter identification. In line with this, our approach minimizes the discrepancy between the model prediction from the reduced model and that of the full model. The remaining parameters in the reduced model might still have large uncertainties, but the correspondence between the model prediction from the reduced model and that of the full model is very good. If required, additional parameter identification could be carried out on the remaining parameters in the reduced model. As the model contains less parameters, parameter identification can be carried out more efficaciously. Note that the parameters that are removed by our method are those that are badly identified, since their absence does not have significant effect on the prediction of the system.

Eventually, we would like to stress that it is always crucial to keep in mind which functionality one wants to preserve in the reduced model. Otherwise, one may arrive at a reduced model that does not serve one's purposes. Therefore, although our reduction method does not require prior biological knowledge, if there is any, that knowledge should always be taken into account.

## Supporting Information

Text S1 **Description of the flower network model in Arabidopsis.**
(PDF)

Figure S1
**The dynamics of the proteins in each four organs.** Measured dynamics are denoted by ‘*’ whereas the dynamics from the reduced model with 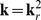 are denoted by the dashed lines. Parameter fitting is applied to dataset of wildtype (a) and knock-out AG (b). The resulted reduced model have a very good prediction for mutants knock-out AP3 (c), knock-out PI (d), ectopically expression of AP3 (e), and ectopically expression of AG (f).(EPS)

Figure S2
**The EGFR biochemical network.** A solid arrow represents a reaction with two kinetic parameters and a dashed arrow represents a reaction with one kinetic parameter. (A) The full network from [25], (B) The optimal network to produce the dynamics of the five target components for any experimental condition 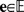 in (27), (C) The optimal network as in (B) but with an additional constraint to maintain the activation pathway to Ras protein.(EPS)

Figure S3
**Model discrimination to distinguish the reduced model with** 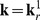 **from the full model with** 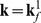 **.** In this case, 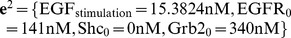. The new dataset obtained from an experiment based on the setting 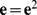 is indicated by ‘*’. The dashed curve in the upper left corner shows that the reduced model cannot fit this dataset.(EPS)

Figure S4
**Result of iterative process to obtain the optimal model for EGFR model.** The threshold value of 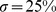 is indicated by the dashed line. For the first dataset, the reduction procedure can remove 33 out of 50 parameters. However, the distance between the reduced and the full models in the first discrimination is still huge, namely 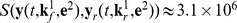. When a new experiment based on experimental condition 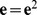 is carried out and the obtained dataset is combined with the first dataset, the number of reduced parameter in the second reduction decreases to 31. Finally, after performing four additional experiments, the distance 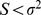, which means that there is no experimental condition that can distinguish the reduced model with 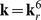 from the full model with 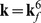. At this stage, the reduced model contains 25 parameters. Since the distance is already smaller than the tolerance, we conclude that the reduced model with 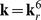 is an optimal model.(EPS)

Figure S5 **Result of iterative process to obtain the optimal model for EGFR model with a constraint to maintain the Ras pathway activation.**
(EPS)

Table S1 **Parameter values of the full and optimal models in the last iteration.** Here the average deviation at each point between the optimal and the full model is less than 25%. Model 1 refers to EGFR model without constraint to prevent the pathway to Ras protein whereas Model 2 refers to EGFR model with the constraint.
(PDF)

Table S2 **List of experiments to obtain optimal model in Model 1.**
(PDF)

Table S3 **List of experiments to obtain optimal model in Model 2.**
(PDF)
