# Supplementary material for: Identifying Optimal Models to Represent Biochemical Systems
Source: PLoS One. 2014 Jan 8;9(1):e83664. doi: 10.1371/journal.pone.0083664 (PMC3885518; doi:10.1371/journal.pone.0083664)
Supplement: Text S1 — Description of the flower network model in Arabidopsis. (PDF) [file pone.0083664.s001.pdf]

Supporting Information

Description of the flower network model

The *A,B,C,D,* and *E* gene types are respectively (*APETALA1 (AP1)*, *3 (AP3), PISTILLATA (PI), AGAMOUS (AG)*, *SHATTERPROOF (SHP)*, and *SEPALLATA (SEP)*, and they are denoted by $x_1 - x_6$. The model in [1] describes monomer dynamics, dimer dynamics, and a mass balance. The mass balance states that the total measured concentrations x^T are the sums of monomer and dimer concentrations.

$$\begin{aligned} x_1^T &= x_1 + 2[x_1x_1] + [x_1x_6] \\ x_2^T &= x_2 + [x_2x_3] \\ x_3^T &= x_3 + [x_2x_3] \\ x_4^T &= x_4 + [x_4x_6] + 2[x_4x_4] \\ x_5^T &= x_5 + [x_5x_6] \\ x_6^T &= x_6 + [x_4x_6] + [x_5x_6] + 2[x_6x_6] + [x_1x_6]. \end{aligned} \tag{1}$$

Here x_i denotes the concentration of monomer i and by $[x_ix_j]$ the concentration of the dimer of proteins i and j. The dynamics of the dimer concentrations consists of the association rate of monomers into dimers, minus the dissociation rate of dimers into monomers. It is assumed that the dimers have a very small, negligible decay.

$$\begin{aligned} \frac{d[x_4x_4]}{dt} &= K_{on,1}x_4x_4 - K_{off,1}[x_4x_4] \\ \frac{d[x_4x_6]}{dt} &= K_{on,2}x_4x_6 - K_{off,2}[x_4x_6] \\ \frac{d[x_5x_6]}{dt} &= K_{on,3}x_5x_6 - K_{off,3}[x_5x_6] \\ \frac{d[x_1x_6]}{dt} &= K_{on,4}x_1x_6 - K_{off,4}[x_1x_6] \\ \frac{d[x_2x_3]}{dt} &= K_{on,5}x_2x_3 - K_{off,5}[x_2x_3] \\ \frac{d[x_1x_1]}{dt} &= K_{on,6}x_1x_1 - K_{off,6}[x_1x_1] \\ \frac{d[x_6x_6]}{dt} &= K_{on,7}x_6x_6 - K_{off,7}[x_6x_6]. \end{aligned} \tag{2}$$

Here K_{on} denotes the association rate, and K_{off} the dissociation rate. The monomer dynamics are as follows

$$\begin{aligned}
\frac{dx_1}{dt} &= \frac{\beta_{1,1}[x_1x_6]}{(K_{1,1}+[x_1x_6])}\frac{Km_{1,2}}{(Km_{1,2}+[x_4x_4])} \\
&\quad -dc_1x_1 - \frac{d[x_1x_6]}{dt} - 2\frac{d[x_1x_1]}{dt} \\
\frac{dx_2}{dt} &= \frac{\beta_{2,1}[x_2x_3]}{Km_{2,1}+[x_2x_3]} + \frac{\beta_{2,2}[x_4x_6]}{Km_{2,2}+[x_4x_6]} \\
&\quad +\frac{\beta_{2,3}[x_1x_6]}{Km_{2,3}+[x_1x_6]} - dc_2x_2 + p_2(t,w) - \frac{d[x_2x_3]}{dt} \\
\frac{dx_3}{dt} &= \frac{\beta_{3,1}[x_2x_3]}{Km_{3,1}+[x_2x_3]} + \frac{\beta_{3,2}[x_4x_6]}{Km_{3,2}+[x_4x_6]} \\
&\quad +\frac{\beta_{3,3}[x_1x_6]}{Km_{3,3}+[x_1x_6]} - dc_3x_3 - \frac{d[x_2x_3]}{dt} \\
\frac{dx_4}{dt} &= \Big(\frac{\beta_{4,1}[x_4x_6]}{Km_{4,1}+[x_4x_6]} + \frac{\beta_{4,2}[x_4x_4]}{Km_{4,2}+[x_4x_4]}\Big)\cdot \\
&\quad \frac{Km_{4,3}}{Km_{4,3}+[x_1x_1]} - dc_4x_4 + p_4(t,w) - \\
&\quad \frac{d[x_4x_6]}{dt} - 2\frac{d[x_4x_4]}{dt} \\
\frac{dx_5}{dt} &= \frac{\beta_{5,1}[x_4x_6]}{(Km_{5,1}+[x_4x_6])}\frac{Km_{5,2}}{(Km_{5,2}+[x_2x_3])} - dc_5x_5 \\
&\quad -\frac{d[x_5x_6]}{dt} \\
\frac{dx_6}{dt} &= \frac{\beta_{6,1}[x_4x_6]}{Km_{6,1}+[x_4x_6]} + \frac{\beta_{6,2}[x_1x_6]}{Km_{6,2}+[x_1x_6]} \\
&\quad +\frac{\beta_{6,3}[x_6x_6]}{Km_{6,3}+[x_6x_6]} - dc_6x_6 - \frac{d[x_4x_6]}{dt} - \\
&\quad \frac{d[x_5x_6]}{dt} - 2\frac{d[x_6x_6]}{dt} - \frac{d[x_1x_6]}{dt} \\
p_2(t,w) &= P_2 \text{ if } w\in[2,3], \text{ and } t\in[1,2] \text{ and elsewhere } 0 \\
p_4(t,w) &= P_4 \text{ if } w\in[3,4], \text{ and } t\in[1,2] \text{ and elsewhere } 0.
\end{aligned} \tag{3}$$

The first fractions on the right hand sides denote activation or repression by Michaelis-Menten kinetics, followed by a decay term. The last terms denote the rates of dimerization. By inserting (3) into (1) it can be verified that the dynamics of the total concentrations only depend on production and decay of the monomers. The p's denote the whorl (w)- and time (t)-dependent trigger mechanisms that are responsible for the different concentration dynamics in each flower whorl. The model consists of 13 state variables (representing proteins and dimers), and 51 parameters representing all the rates of the biochemical interactions.

Model (1)–(3) can be simplified using physical arguments, via a quasi-steady state assumption of the relatively fast dimer dynamics. This simplification was also used in [1] for parameter estimation purposes. The dimer equations (2) then take the form

$$\begin{aligned}
[x_4x_4] &= \gamma_1 x_4^2 \\
[x_4x_6] &= \gamma_2 x_4 x_6 \\
[x_5x_6] &= \gamma_3 x_5 x_6 \\
[x_1x_6] &= \gamma_4 x_1 x_6 \\
[x_2x_3] &= \gamma_5 x_2 x_3 \\
[x_1x_1] &= \gamma_6 x_1^2 \\
[x_6x_6] &= \gamma_7 x_6^2,
\end{aligned} \tag{4}$$

with $\gamma = \frac{K_{on}}{K_{off}}$, and these are inserted into (3), using the chain rule

$$\frac{d[x_i x_j]}{dt} = \gamma\Big(\frac{dx_i}{dt} x_j + x_i \frac{dx_j}{dt}\Big). \tag{5}$$

This gives an ODE model for the monomer dynamics of the form

$$\frac{dx}{dt} = F + B\frac{dx}{dt}, \tag{6}$$

with

$$F = \begin{pmatrix} \frac{\frac{\beta_{1,1}\gamma_4 x_1 x_6}{(K_{1,1}+\gamma_4 x_1 x_6)} \frac{Km_{1,2}}{(Km_{1,2}+\gamma_1 x_4^2)} - dc_1 x_1}{1+\gamma_4 x_6 + 4\gamma_1 x_1} \\ \frac{\frac{\beta_{2,1}\gamma_5 x_2 x_3}{Km_{2,1}+\gamma_5 x_2 x_3} + \frac{\beta_{2,2}\gamma_2 x_4 x_6}{Km_{2,2}+\gamma_2 x_4 x_6} + \frac{\beta_{2,3}\gamma_4 x_1 x_6}{Km_{2,3}+\gamma_4 x_1 x_6} - dc_2 x_2 + p_2(t,w)}{1+\gamma_5 x_3} \\ \frac{\frac{\beta_{3,1}\gamma_5 x_2 x_3}{Km_{3,1}+\gamma_5 x_2 x_3} + \frac{\beta_{3,2}\gamma_2 x_4 x_6}{Km_{3,2}+\gamma_2 x_4 x_6} + \frac{\beta_{3,3}\gamma_4 x_1 x_6}{Km_{3,3}+\gamma_4 x_1 x_6} - dc_3 x_3}{1+\gamma_5 x_2} \\ \frac{\left(\frac{\beta_{4,1}\gamma_2 x_4 x_6}{Km_{4,1}+\gamma_2 x_4 x_6} + \frac{\beta_{4,2}\gamma_1 x_4^2}{Km_{4,2}+\gamma_1 x_4^2}\right) \cdot \frac{Km_{4,3}}{Km_{4,3}+\gamma_6 x_1^2} - dc_4 x_4 + p_4(t,w)}{1+\gamma_2 x_6 + 4\gamma_1 x_4} \\ \frac{\frac{\beta_{5,1}\gamma_2 x_4 x_6}{(Km_{5,1}+\gamma_2 x_4 x_6)} \frac{Km_{5,2}}{(Km_{5,2}+\gamma_5 x_2 x_3)} - dc_5 x_5}{1+\gamma_3 x_6} \\ \frac{\frac{\beta_{6,1}\gamma_2 x_4 x_6}{Km_{6,1}+\gamma_2 x_4 x_6} + \frac{\beta_{6,2}\gamma_4 x_1 x_6}{Km_{6,2}+\gamma_4 x_1 x_6} + \frac{\beta_{6,3}\gamma_7 x_6^2}{Km_{6,3}+\gamma_7 x_6^2} - dc_6 x_6}{1+\gamma_2 x_4 + 4\gamma_7 x_6 + \gamma_4 x_1 + \gamma_3 x_5} \end{pmatrix} \tag{7}$$

and

$$B = -\begin{pmatrix} 0 & 0 & 0 & 0 & 0 & \frac{\gamma_4 x_1}{1+\gamma_4 x_6 + 4\gamma_1 x_1} \\ 0 & 0 & \frac{\gamma_5 x_2}{1+\gamma_5 x_3} & 0 & 0 & 0 \\ 0 & \frac{\gamma_5 x_3}{1+\gamma_5 x_2} & 0 & 0 & 0 & 0 \\ 0 & 0 & 0 & 0 & 0 & \frac{\gamma_2 x_4}{1+\gamma_2 x_6 + 4\gamma_1 x_4} \\ 0 & 0 & 0 & 0 & 0 & \frac{\gamma_3 x_5}{1+\gamma_3 x_6} \\ \frac{\gamma_4 x_6}{n_6} & 0 & 0 & \frac{\gamma_2 x_6}{n_6} & \frac{\gamma_3 x_6}{n_6} & 0 \end{pmatrix}, \tag{8}$$

with $n_6 = 1 + \gamma_2 x_4 + 4\gamma_7 x_6 + \gamma_4 x_1 + \gamma_3 x_5$. For numerical simulation, equation (6) can be written in explicit ODE form as

$$\frac{dx}{dt} = (I - B)^{-1} F. \tag{9}$$

The output consisting of protein concentrations that are part of dimers, x^D, can be found in the mass balance by subtracting the monomer concentrations from the total concentrations, using (4):

$$\begin{aligned} x_1^D &= 2\gamma_6 x_1^2 + \gamma_4 x_1 x_6 \\ x_2^D &= \gamma_5 x_2 x_3 \\ x_3^D &= \gamma_5 x_2 x_3 \\ x_4^D &= \gamma_2 x_4 x_6 + 2\gamma_1 x_4^2 \\ x_5^D &= \gamma_3 x_5 x_6 \\ x_6^D &= \gamma_2 x_4 x_6 + \gamma_3 x_5 x_6 + 2\gamma_7 x_6^2 + \gamma_4 x_1 x_6. \end{aligned} \tag{10}$$

Model (9) with output (10) now consists of monomers, and is reduced to 6 dynamic equations and 44 parameters. Based on biological knowledge, see [1], the parameter values for the association and dissociation event, K_{on} and K_{off}, are fixed so that the corresponding γ also becomes fixed, and hence we are left with 37 parameters to be estimated.

Supplementary Figures

Figure S1. The dynamics of the proteins in each four organs. Measured dynamics are denoted by '*' whereas the dynamics from the reduced model with $\mathbf{k} = \mathbf{k}_r^2$ are denoted by the dashed lines. Parameter fitting is applied to dataset of wildtype (a) and knock-out AG (b). The resulted reduced model have a very good prediction for mutants knock-out AP3 (c), knock-out PI (d), ectopically expression of AP3 (e), and ectopically expression of AG (f).

Figure S2. The EGFR biochemical network. A solid arrow represents a reaction with two kinetic parameters and a dashed arrow represents a reaction with one kinetic parameter. (A) The full network from [2], (B) The optimal network to produce the dynamics of the five target components for any experimental condition $\mathbf{e} \in \mathbb{E}$ in (27), (C) The optimal network as in (B) but with an additional constraint to maintain the activation pathway to Ras protein.

Figure S3. Model discrimination to distinguish the reduced model with $\mathbf{k} = \mathbf{k}_r^1$ from the full model with $\mathbf{k} = \mathbf{k}_f^1$. In this case, $\mathbf{e}^2 = \{\text{EGF}_{\text{stimulation}} = 15.3824$ nM, $\text{EGFR}_0 = 141$ nM, $\text{Shc}_0 = 0$ nM, $\text{Grb2}_0 = 340$ nM$\}$. The new dataset obtained from an experiment based on the setting $\mathbf{e} = \mathbf{e}^2$ is indicated by '*'. The dashed curve in the upper left corner shows that the reduced model cannot fit this dataset.

Figure S4. Result of iterative process to obtain the optimal model for EGFR model. The threshold value of $\sigma = 25\%$ is indicated by the dashed line. For the first dataset, the reduction procedure can remove 33 out of 50 parameters. However, the distance between the reduced and the full models in the first discrimination is still huge, namely $S(\mathbf{y}(t, \mathbf{k}_f^1, \mathbf{e}^2), \mathbf{y}_r(t, \mathbf{k}_r^1, \mathbf{e}^2)) \approx 3.1 \times 10^6$. When a new experiment based on experimental condition $\mathbf{e} = \mathbf{e}^2$ is carried out and the obtained dataset is combined with the first dataset, the number of reduced parameter in the second reduction decreases to 31. Finally, after performing four additional experiments, the distance $S < \sigma^2$, which means that there is no experimental condition that can distinguish the reduced model with $\mathbf{k} = \mathbf{k}_r^6$ from the full model with $\mathbf{k} = \mathbf{k}_f^6$. At this stage, the reduced model contains 25 parameters. Since the distance is already smaller than the tolerance, we conclude that the reduced model with $\mathbf{k} = \mathbf{k}_r^6$ is an optimal model.

Figure S5. Result of iterative process to obtain the optimal model for EGFR model with a constraint to maintain the Ras pathway activation.

Supplementary Tables

Table S1. Parameter values of the full and optimal models in the last iteration. Here the average deviation at each point between the optimal and the full model is less than 25%. Model 1 refers to EGFR model without constraint to prevent the pathway to Ras protein whereas Model 2 refers to EGFR model with the constraint.

Table S2. List of experiments to obtain optimal model in Model 1.

Table S3. List of experiments to obtain optimal model in Model 2.

References

[1] van Mourik S, van Dijk AD, de Gee M, Immink RG, Kaufmann K, et al. (2010) Continuous-time modeling of cell fate determination in Arabidopsis flowers. BMC Syst Biol 4: 101.

[2] Kholodenko BN, Demin OV, Moehren G, Hoek JB (1999) Quantification of Short Term Signaling by the Epidermal Growth Factor Receptor. Journal of Biological Chemistry 274: 30169-30181.
