# Supplementary material for: Identifying Optimal Models to Represent Biochemical Systems
Source: PLoS One. 2014 Jan 8;9(1):e83664. doi: 10.1371/journal.pone.0083664 (PMC3885518; doi:10.1371/journal.pone.0083664)
Supplement: Table S1 — Parameter values of the full and optimal models in the last iteration. Here the average deviation at each point between the optimal and the full model is less than 25%. Model 1 refers to EGFR model without constraint to prevent the pathway to Ras protein whereas Model 2 refers to EGFR model with the constraint. (PDF) [file pone.0083664.s007.pdf]

Table S1: Parameter values of the full and optimal models in the last iteration. Here the average deviation at each point between the optimal and the full model is less than 25%. Model 1 refers to EGFR model without constraint to prevent the pathway to Ras protein whereas Model 2 refers to EGFR model with the constraint.

Parameter	Model 1		Model 2	
	$\mathbf{k}_f^6$	$\mathbf{k}_r^6$	$\mathbf{k}_f^7$	$\mathbf{k}_r^7$
k_1	3.1×10^{-3}	2.8×10^{-3}	3×10^{-3}	2.7×10^{-3}
k_{-1}	5.77×10^{-2}	5.31×10^{-2}	5.84×10^{-2}	5.09×10^{-2}
k_2	9.5×10^{-3}	9.9×10^{-3}	9.6×10^{-3}	9.8×10^{-3}
k_{-2}	0.1336	9.24×10^{-2}	8.22×10^{-2}	0.1087
k_3	1.0679	0.79	0.9481	1.0264
k_{-3}	0.0194	0	0.016	0
V_4	450.0001	477.8907	440.9235	437.1619
K_4	49.9990	48.2002	51.0252	46.1166
k_5	6.57×10^{-2}	5.95×10^{-2}	6.4×10^{-2}	5.1×10^{-2}
k_{-5}	0.1917	0	0.3116	0
k_6	0.9992	1.0237	0.9264	0.8975
k_{-6}	4.41×10^{-2}	0	4.04×10^{-2}	0
k_7	0.3079	0.2803	0.3053	0.2852
k_{-7}	4×10^{-3}	7.4×10^{-3}	5×10^{-3}	4.6×10^{-3}
V_8	0.9894	0	0.7757	0
K_8	100	112.7967	101.4729	97.2425
k_9	4.2×10^{-3}	4.4×10^{-3}	2.8×10^{-3}	2.7×10^{-3}
k_{-9}	7.95×10^{-2}	4.03×10^{-2}	4.45×10^{-2}	3.19×10^{-2}
k_{10}	5.8×10^{-3}	0	4.9×10^{-3}	0
k_{-10}	5.26×10^{-2}	0	4.05×10^{-2}	0
k_{11}	7.68×10^{-2}	0	9.18×10^{-2}	0
k_{-11}	1.66×10^{-2}	0	1.64×10^{-2}	0
k_{12}	1.73×10^{-2}	0	7.5×10^{-3}	0
k_{-12}	1.073×10^{-4}	0	5.07×10^{-5}	0
k_{13}	9.48×10^{-2}	0.1036	8.49×10^{-2}	8.39×10^{-2}
k_{-13}	0.6009	0	0.6059	0
k_{14}	5.9984	1×10^{4}	5.3676	1×10^{4}
k_{-14}	5.63×10^{-2}	0	9.71×10^{-2}	0
k_{15}	0.3346	0.2904	0.3024	0.2903
k_{-15}	1×10^{-3}	2.6×10^{-3}	1.6×10^{-3}	1.1×10^{-3}
K_{16}	1.6962	1.9833	1.7624	1.6462
V_{16}	340	330.3266	324.2768	327.9718
k_{17}	2.9×10^{-3}	2.8×10^{-3}	3.4×10^{-3}	2.7×10^{-3}
k_{-17}	0.1043	0	8.53×10^{-2}	0
k_{18}	0.2973	0.2272	0.2962	0.2016
k_{-18}	4×10^{-4}	1.6×10^{-3}	2.54×10^{-5}	0
k_{19}	1.83×10^{-2}	0	1.65×10^{-2}	0
k_{-19}	2.25×10^{-2}	0	0.1027	0
k_{20}	0.1235	0	0.1159	0.1928
k_{-20}	8.496×10^{-4}	0	2.596×10^{-4}	6.813×10^{-4}
k_{21}	2.7×10^{-3}	3.1×10^{-3}	2.8×10^{-3}	2.4×10^{-3}
k_{-21}	9.84×10^{-2}	3.54×10^{-2}	9.72×10^{-2}	7.51×10^{-2}
k_{22}	2.95×10^{-2}	0	7.48×10^{-2}	9.6×10^{-3}
k_{-22}	5.35×10^{-2}	0	1.38×10^{-2}	0.1298
k_{23}	9.66×10^{-2}	0	0.1307	0
k_{-23}	2.99×10^{-2}	0	4.13×10^{-2}	0
k_{24}	1.24×10^{-2}	0	1.41×10^{-2}	0
k_{-24}	4.86×10^{-2}	0	8.14×10^{-2}	0

Continued on next page

Table S1 – *Continued from previous page*

Parameter	Model 1		Model 2	
	$\mathbf{k}_f^6$	$\mathbf{k}_r^6$	$\mathbf{k}_f^7$	$\mathbf{k}_r^7$
k_{25}	1.0068	1.1123	0.9916	1.0597
k_{-25}	3.36×10^{-2}	3.41×10^{-2}	3.12×10^{-2}	3.43×10^{-2}
