# Supplementary material for: Identifying Optimal Models to Represent Biochemical Systems
Source: PLoS One. 2014 Jan 8;9(1):e83664. doi: 10.1371/journal.pone.0083664 (PMC3885518; doi:10.1371/journal.pone.0083664)
Supplement: Table S2 — List of experiments to obtain optimal model in Model 1. (PDF) [file pone.0083664.s008.pdf]

Table S2: List of experiments to obtain optimal model in Model 1.

Experiment index	Experimental condition ($\mathbf{e}$)			
	$EGF_{stimulation}$ (nM)	$EGFR_0$ (nM)	Shc_0 (nM)	$Grb2_0$ (nM)
1a	20	100	150	85
1b	2	100	150	85
2	15.3824	141	340	0
3	0.0746	2.5771	156.2478	10.4315
4	6.5245	26.4265	0.7839	0.5163
5	0.0118	400	6.2924	150
6	19.9934	399.9985	0.0506	150
