# Supplementary material for: Identifying Optimal Models to Represent Biochemical Systems
Source: PLoS One. 2014 Jan 8;9(1):e83664. doi: 10.1371/journal.pone.0083664 (PMC3885518; doi:10.1371/journal.pone.0083664)
Supplement: Table S3 — List of experiments to obtain optimal model in Model 2. (PDF) [file pone.0083664.s009.pdf]

Table S3: List of experiments to obtain optimal model in Model 2.

Experiment index	Experimental condition ($\mathbf{e}$)			
	$EGF_{stimulation}$ (nM)	$EGFR_0$ (nM)	Shc_0 (nM)	$Grb2_0$ (nM)
1a	20	100	150	85
1b	2	100	150	85
2	20	400	340	0
3	0.0653	2.1885	0	11.3049
4	0.0936	3.1826	340	150
5	20	0.4287	107.2116	149.9955
6	20	400	0.7385	0.8746
7	20	239.0186	0.1460	149.9980
